# Anorectal Manometry in Pediatric Colorectal Surgical Care

**DOI:** 10.3390/children11060654

**Published:** 2024-05-28

**Authors:** Justin C. Wheeler, Scott S. Short, Michael D. Rollins

**Affiliations:** 1 Division of Pediatric Gastroenterology, University of Utah, Salt Lake City, UT 84113, USA; justin.wheeler@hsc.utah.edu; 2 Division of Pediatric Surgery, University of Utah, Salt Lake City, UT 84112, USA

**Keywords:** pediatric, colorectal surgery, anorectal manometry, constipation, dyssynergia, pelvic rehabilitation, anorectal malformation, Hirschsprung disease

## Abstract

Background: Pediatric colorectal specialists care for patients with a variety of defecation disorders. Anorectal (AR) manometry testing is a valuable tool in the diagnosis and management of these children. This paper provides a summary of AR manometry techniques and applications as well as a review of AR manometry findings in pediatric patients with severe defecation disorders referred to a pediatric colorectal center. This is the first study describing multi-year experience using a portable AR manometry device in pediatric patients. Methods: An electronic medical record review was performed (1/2018 to 12/2023) of pediatric patients with defecation disorders who had AR manometry testing. Demographics, diagnostic findings, and outcomes are described. Key Results: A total of 297 unique patients (56.9% male, *n* = 169) had AR manometry testing. Of these, 72% (n = 188) had dyssynergic defecation patterns, of which 67.6% (n = 127) had fecal soiling prior to treatment. Pelvic rehabilitation (PR) was administered to 35.4% (n = 105) of all patients. A total of 79.5% (n = 58) of the 73 patients that had fecal soiling at initial presentation and completed PR with physical therapy and a bowel management program were continent after therapy. AR manometry was well tolerated, with no major complications. Conclusions: AR manometry is a simple test that can help guide the management of pediatric colorectal surgical patients with defecation disorders. As a secondary finding, PR is a useful treatment for patients with dyssynergic stooling.

## 1. Introduction

Children with a variety of defecation disorders may be referred to a pediatric colorectal center for care. Anorectal (AR) manometry can be a valuable tool in the diagnostic work-up and management of pediatric patients with disordered defecation, ranging from relatively common conditions including functional constipation, fecal soiling, rectal prolapse, screening for Hirschsprung disease (HD), post-surgical care of HD, anorectal malformations, traumatic perineal injuries, and/or spinal anomalies [1,2,3,4,5,6]. This article describes the basics of anorectal manometry testing and its application in pediatric colorectal surgical care and highlights a 6-year experience with anorectal manometry at a single pediatric colorectal center. 

## 2. Anorectal Manometry Basics

### 2.1. What Is Anorectal Manometry?

**Figure 1:**
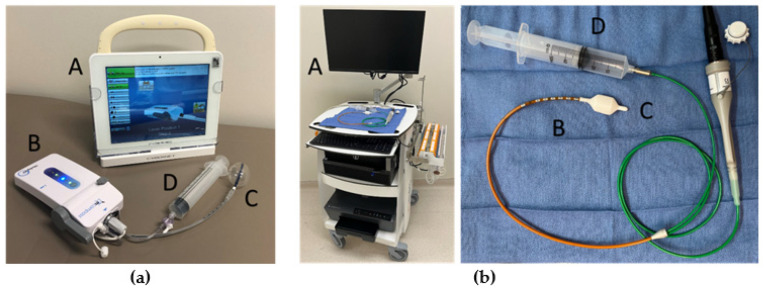
Examples of anorectal manometry systems. (**a**) Portable anorectal manometry system: (mCompass, Medspira), with a tablet computer display screen (A), wireless manometer (B), disposable catheter with four small radially oriented balloons that span the anal canal and a single rectal balloon (C), and a syringe to inflate and deflate the rectal balloon (D). (**b**) High-resolution (HR) anorectal manometry system: (Solar GI, Laborie) with a monitor display screen, manometer processor, and computer tower (A), solid-state manometry catheter with nine radially oriented sensors (B), a disposable rectal balloon (C), and a syringe to inflate and deflate the rectal balloon (D).

AR manometry is a minimally invasive test that can be used to characterize anal sphincter function, rectal sensation thresholds, and defecation dynamics [7]. It is the most commonly performed manometry study in pediatric patients. AR manometry testing is often limited to large referral centers due to relatively few trained pediatric providers with expertise in test interpretation and the high cost of purchasing and maintaining manometry equipment [5]. In recent years, portable AR manometry systems using wireless technology and disposable catheters have become available [8]. The test uses a thin catheter with pressure sensors that span the anal canal and an inflatable rectal balloon on the end that measures rectal pressure (Figure 1). The test is usually performed with an awake and cooperative patient, although portions of the test can be performed on a sedated individual. AR manometry testing typically includes measuring anal sphincter pressure at rest, maximum anal sphincter pressures with a volitional anal sphincter squeeze, sensation thresholds, rectal compliance to rectal distension with an air-filled balloon, defecation dynamics with simulated stooling, and screening for the presence of normal anorectal reflexes (e.g., a cough reflex or a rectoanal inhibitory reflex (RAIR)). An absent RAIR may be indicative of HD or anal achalasia [9]. 

### 2.2. Anorectal Manometry Testing

There is some variation between centers on how AR manometry testing is performed. Several AR manometry testing protocols have been proposed over the years in an attempt to standardize testing and reporting. The most recent protocol, the London Protocol, has become common but it has not yet been validated specifically for pediatric patients [10,11]. Patients receive a pediatric saline enema on the day of the test to ensure an empty rectal vault at the time of the study. The study can be performed by a physician or an appropriately trained team member. Child life specialists should be available for age-appropriate education and distraction before and during the study. Testing typically takes 15–20 min to complete but can vary due to patient cooperation. The test is often performed in a procedure center, although bedside testing in a clinic setting can also be performed with some patients.

**Figure 2:**
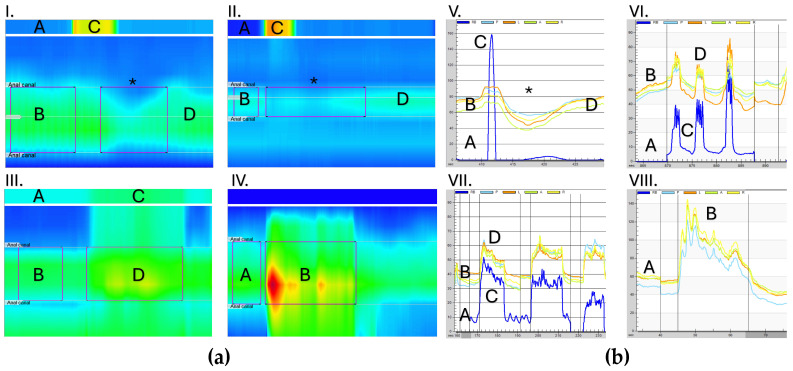
Examples of anorectal manometry tracings. (**a**) High-resolution (HR) AR manometry Clouse plots: I. and II. Normal rectoanal inhibitory reflex (RAIR) in an awake (I) and sedated (II) patient. Rectal (A) and anal sphincter pressure (B) at rest, a rapid increase in rectal pressure with rectal balloon inflation (C) and then deflation, followed by a reflexive drop in anal sphincter pressure (*) and a return to baseline anal sphincter pressure (D). Lower anal sphincter resting pressure (B) is expected in the sedated patient (II). III. Dyssynergic stooling pattern. Normal rectal (A) and anal sphincter (B) pressures at rest, followed by an expected increase in rectal pressure when bearing down (C), but accompanied by a paradoxical increase in anal sphincter pressure (D) rather than relaxation. This may contribute to ineffective defecation. IV. Anal sphincter squeeze. Normal anal sphincter pressure at rest (A) followed by an increase in anal sphincter pressure when instructed to squeeze (B). A component of fatigue is present characterized by a slow drop in sphincter pressure over time. (**b**) Portable AR manometry line tracings: V. Normal rectoanal inhibitory reflex (RAIR): Rectal (A, dark blue line) and anal sphincter (B, four multi-color lines representing four radially oriented air-charged manometry balloons) pressures at rest, and a rapid increase in rectal pressure with rectal balloon inflation (C) and then deflation, followed by a reflexive drop in anal sphincter pressure (*) and a return to baseline anal sphincter pressure (D). VI. Cough Reflex: Rectal (A) and anal sphincter (B) pressures at rest, with an increase in rectal pressure (C) and an expected normal reflexive increase in anal sphincter pressure (D) during a simulated cough. This is a normal physiologic response to increases in intrabdominal pressure to prevent stress incontinence. VII. Dyssynergic stooling pattern: Rectal (A) and anal sphincter pressure (B) at rest, with an adequate increase in pressure when pushing (C) but a paradoxical increase in anal sphincter pressure (D), mirroring the increase in rectal pressure rather than relaxation. VIII. Anal sphincter volitional squeeze: The normal anal sphincter pressure at rest (A) followed by an increase in anal sphincter pressure when instructed to squeeze (B). A component of fatigue is present, characterized by a drop in sphincter pressure over time.

Testing parameters may include (a) resting pressure, (b) endurance squeeze, (c) maximum squeeze, (d) push attempts, (e) rectal sensation and rectal compliance testing, (f) rectoanal inhibitory reflex (RAIR), (g) cough reflex, and (h) catheter expulsion. Following the conclusion of each study, the tracings are reviewed and reported by a pediatric gastroenterologist trained in manometry interpretation [11,12,13,14]. High-resolution (HR) AR manometry data are often presented using color-based pressure topography mapping known as a Clouse plot, which uses a process of averaging pressures between sensors to display results of pressure over a period of time in seamless isobaric color regions (Figure 2) [15]. Non-HR manometry tracings are presented as linear tracings of pressure over time (Figure 2). Ranges of normal values have been published for women, men, and pediatric patients [12,16]. 

Potential abnormal AR manometry findings could include hyper- or hypotonic anal sphincter resting pressures, hypocontractile anal sphincter squeeze, uncoordinated (dyssynergic) defecation mechanics, hyper- or hyposensitive rectal sensation thresholds, an absent RAIR, and/or an absent cough reflex. An abnormal AR manometry result can be indicative of a number of conditions and subsequently guide further management. 

Defecation dynamics can be characterized by examining rectal and anal sphincter pressure and function during simulated defecation. Simulated defecation during AR manometry testing involves a patient bearing down and pushing. Uncoordinated defecation is known as dyssynergia. Dyssynergic defecation is characterized by paradoxical anal contraction or inadequate increase in rectal pressures during attempted defecation [17]. There is evidence that children with dyssynergic stooling treated with pelvic floor rehabilitation can improve stooling patterns [18,19,20].

## 3. AR Manometry in Pediatric Colorectal Surgical Care

### 3.1. Constipation 

The differential for a pediatric patient with constipation is broad, with functional constipation (slow colonic transit, behavioral withholding, diet-related) accounting for up to 95% of causes in pediatric patients [2]. The majority of children with constipation can be treated effectively in a primary care setting with dietary modification, oral laxatives, and/or behavior modification [21]. Children who fail routine management may be referred to pediatric colorectal specialists for additional diagnostic work-up and management. Many colorectal specialists, including pediatric colorectal surgeons, advocate for the initiation of defined bowel management programs. Common strategies include high-dose stimulant laxatives or large-volume enemas (LVEs) [22]. AR manometry can be an effective tool integrated into a bowel management program. Eliciting a RAIR can effectively rule out HD or anal achalasia, eliminating the need for a rectal biopsy if the provider has concerns for HD [9]. Conversely, an absent RAIR would support proceeding with a rectal biopsy. Patients with chronic constipation can develop a dilated rectum, leading to the inability to sense stool until there is a large rectal stool burden. This can be associated with overflow incontinence or soiling. AR manometry testing can establish rectal sensation thresholds while providing objective data for patient caregivers and providers to characterize a patient’s condition and improve caregiver buy-in for recommended interventions like high-dose stimulant laxatives, enema therapy, or pelvic rehabilitation. 

More subtle findings, such as dyssynergic stooling, can be identified with AR manometry testing, which may contribute to constipation due to ineffective evacuation (Figure 2). Patients with dyssynergic stooling can be referred to an experienced pelvic floor physical therapist. Limited data suggests that biofeedback may be more effective than laxatives in patients with dyssynergia and normal transit constipation [23,24]. 

AR manometry can support clinical decision making when considering a transition in constipation management from large-volume enemas to laxatives or weaning off of laxatives completely. Screening for a return of rectal sensation and normal defecation dynamics may support decision making by providers and families [6]. A persistent lack of rectal sensation may support postponing a change in management to allow for further recovery of rectal tone and sensation with ongoing bowel management and pelvic rehabilitation. Behavioral health interventions should also be employed without hesitancy for children struggling with chronic defecation challenges, as there are often overlying psychosocial factors contributing to symptoms [21]. 

### 3.2. Fecal Soiling

Fecal soiling affects 1.1–4.4% of children and can be a significant source of stress for parents and children [1,25]. The most common cause of fecal soiling in toilet-trained children is overflow incontinence in the setting of withholding stool, also known as encopresis [26]. This functional disorder is attributed to liquid stool leaking around a formed fecal mass and passing out of the anal canal when the anal sphincter relaxes. The internal anal sphincter complex is the largest contributing factor to passive fecal continence [27]. Any condition with decreased or absent anal sphincter tone or poor rectal sensation will increase the risk of fecal incontinence. This includes patients with anorectal malformations, perineal or anal trauma, or spinal injury or anomalies. 

The AR manometry metric with the most sensitivity and specificity for discriminating between continent and incontinent individuals is anal squeeze pressure, although the correlation is imperfect [28]. The finding of hypotensive anal sphincter resting tone on AR manometry testing is supportive of an increased risk of stress incontinence, particularly if rectal pressures surpass the reflexive increase in anal sphincter pressure during a simulated cough. The cough reflex remains intact for spinal lesions above the sacrum but may be absent in sacral spinal lesions like a tethered cord or cauda equina syndrome or with pudendal nerve injury [29]. An absent cough reflex during AR manometry testing would support additional diagnostic work-up with magnetic resonance imaging (MRI) of the lumbar spine to screen for spinal abnormalities. AR manometry can also be used when considering transitioning patients with functional fecal incontinence from large-volume enemas to oral laxatives to see if sensation and anal sphincter function are improved or intact [6]. 

### 3.3. Rectal Prolapse

Rectal prolapse is the extrusion of some or all of the rectal mucosa through the anal sphincter and is a common and typically self-limited occurrence in pediatric patients [30]. Idiopathic rectal prolapse is commonly attributed in the United States to constipation or straining when defecating. Other causes of prolapse should be considered, including diarrheal illness, parasite infection, proctitis from anal insertion of foreign objects, solitary rectal ulcer, anal sphincter injury, cystic fibrosis, celiac disease, or a lead point like a polyp [30]. In addition to medical management using high-dose stimulant laxatives and behavioral interventions, AR manometry can be used to identify patients with dyssynergic stooling patterns and guide referral to pelvic rehabilitation with an experienced physical therapist. A typical dyssynergic pattern could entail very high abdominal pushing attempts with inadequate anal sphincter relaxation (Figure 2). When idiopathic rectal prolapse is diagnosed, most patients will have a resolution of symptoms with appropriate bowel management and behavioral changes [31]. Surgical interventions such as rectopexy are reserved for refractory and persistent prolapse. 

### 3.4. Hirschsprung Disease

Hirschsprung disease (HD) is a congenital disease characterized by the absence of ganglion cells in the myenteric and submucosal plexuses of the bowel, resulting in functional bowel outlet obstruction [32]. HD is typically diagnosed with a rectal suction biopsy (RSB) or full-thickness rectal biopsy [9]. Rectal biopsy is considered the gold standard for the diagnosis of HD. However, AR manometry can be used as an effective screening tool and may avoid an operative procedure. Patients with HD will have an absent RAIR on AR manometry testing [3,33]. When a RAIR is present, HD can effectively be excluded. However, a false-positive RAIR can occur if there is displacement of the sensor spanning the internal anal sphincter during balloon inflation or with relaxation of the external anal sphincter rather than the internal anal sphincter [2]. The use of AR manometry as the initial diagnostic test in infants with concern for HD may decrease the number of rectal suction biopsies required for diagnosis [9]. RSB requires repeat biopsy in around 10% of patients due to inadequate tissue or inconclusive results but has around 96% sensitivity and 99% specificity for HD if performed properly [34]. In comparison, one systematic review showed AR manometry has 91% specificity and 94% sensitivity when screening for HD [35]. After surgical repair, a RAIR will remain absent due to abnormal innervation of the anal sphincter complex. 

Dysfunctional stooling, such as persistent constipation and fecal soiling, may occur in patients with HD after initial surgical resection of the aganglionic bowel [36]. This can be due to a number of etiologies, and a systematic work-up may include screening for anastomotic stricture, a twisted pull-through, obstructing seromuscular cuff (if underwent a Yancey–Soave repair), dysfunctional Duhamel pouch, poor intrinsic colonic motility, anal sphincter spasm, or residual aganglionic bowel [37]. AR manometry testing can identify high anal sphincter tone or spasm or dyssynergic stooling patterns. Lack of sensation could be indicative of damage to the dentate line, poor sensation in the neo-rectum, or development of a dilated and insensate rectum from withholding behaviors. Low anal sphincter pressure could signal prior anal sphincter damage during the pull-through procedure. Interventions could include formal bowel management with pelvic rehabilitation. Patients with high sphincter tone or other obstructive symptoms may benefit from anal sphincter botulinum toxin A injections [38]. 

### 3.5. Anorectal Malformation and Trauma

Following the repair of ARM, patients may struggle with either constipation or fecal soiling due to the lack of a typical anal sphincter complex. AR manometry testing can demonstrate if high or low neo-anal tone is present and the degree of compensatory pelvic floor function utilized by a patient to support continence. It can also provide objective data for the risk of stress incontinence by comparing neo-anal sphincter pressure to rectal pressures during a purposeful cough or Valsalva maneuver. Families and providers may use these data to give a clearer picture of the potential for soiling when considering a change in therapy, such as transitioning from enema to laxative therapy. 

Traumatic injury to the perineum or anal sphincter complex can result in ineffective anal sphincter function [28]. Some patients with traumatic injury may require a diverting colostomy to protect the surgical repair. AR manometry can characterize anal sphincter pressure and response to volitional squeeze in a cooperative patient following repair, which provides a dynamic, live picture of function compared to sedated electrical perianal muscle stimulation. Commonly available HR AR manometry catheters can capture a degree of circumferential anatomic function, including creating a 3D representation of the anal canal, although with less detail than a high-definition AR manometry catheter [39]. These results can contribute to care discussions such as the timing of colostomy closure, the potential for continence, or the future need for retrograde or antegrade enema therapy following colostomy closure. 

### 3.6. AR Manometry Testing Limitations and Challenges

AR manometry testing has a number of limitations. AR manometry can be embarrassing and socially uncomfortable for children and teenagers. Having a child life specialist present for testing to address patient anxiety and to help with education and distraction can help obviate these concerns. Testing requires subjective patient reporting of sensation and a great deal of patient cooperation to evaluate stooling dynamics. Children must be at least 6 years old to perform the test in the clinic. Younger patients are less likely to provide needed sensory feedback, so AR manometry testing in younger age groups primarily is to screen for an intact RAIR and anal sphincter resting pressures. As such, we typically perform HR-AR manometry in our procedure center for any children under 6 years old, often under sedation. Patients who require sedation can be sedated with ketamine or propofol while avoiding gases like sevoflurane, which can dampen the RAIR response [40]. 

The clinical significance of dyssynergic stooling patterns on AR manometry is controversial. The reported prevalence of dyssynergia in patients with chronic constipation varies widely, with 20–81% of adult patients who underwent AR manometry testing in tertiary referral centers being found to have dyssynergia [17,41]. Abnormal stooling patterns suggestive of dyssynergia can be present in up to 20% of asymptomatic adult patients [42]. Factors such as non-physiologic positioning on the exam table during expulsion attempts, embarrassment about defecating while expelling the balloon during simulated defecation, or difficulty understanding expulsion instructions could produce a high false positive rate. The recently published guidelines by the international anorectal physiology working group known as the London Classification classifies dyssynergic stooling patterns on AR manometry as being a minor or inconclusive finding and recommends AR manometry testing only if a patient fails a balloon expulsion test [11]. The published literature has been mixed on the efficacy of pelvic floor rehabilitation for children with dyssynergia, but many reports have shown excellent outcomes with improved stool frequency, a decrease in laxative doses, and decreased soiling [18,19,23,24,43]. The efficacy may be due in part to the frequent nature of follow-up appointments, which helps reinforce compliance with therapies and behavior modification that cannot be accomplished with typically infrequent clinic visits. Therefore, findings of dyssynergic patterns on manometry should be interpreted in conjunction with clinical symptoms. The use of additional testing modalities of defecation dynamics, such as the balloon expulsion test or scintigraphic defecography, could be considered [14,44].

## 4. Methods and Materials 

### 4.1. The Utah Pediatric Colorectal Center Experience with Anorectal Manometrys

The Pediatric Colorectal Center at Primary Children’s Hospital is a multidisciplinary center that manages pediatric patients with defecation disorders. This colorectal center includes a constipation, Hirschsprung, and anorectal malformation clinic directed by a pediatric surgical team, an inflammatory bowel disease clinic, and a motility disorders clinic directed by a pediatric gastroenterology motility specialist. To describe our experience using AR manometry to manage pediatric patients with defecation disorders, we performed a retrospective chart review of patients ages 0–19 years who underwent AR manometry testing as part of their evaluation between 1/2018 and 12/2023. This study was approved by the University of Utah Institutional Review Board, reference number IRB_00119069. Patients who had AR manometry testing were identified from an existing database maintained to track outcomes. Diagnoses included functional constipation as defined by the Rome IV criteria [45], fecal soiling, rectal prolapse, refractory constipation or fecal soiling in the setting of post-surgical HD, spinal anomalies, trauma, and anorectal malformations (Table 1). 

The primary objective of this study was to describe how AR manometry testing guides clinical management in colorectal center patients. Secondary outcomes of the study were examining the clinical course of patients with fecal soiling managed with bowel management protocols and the utility of physical therapy for PR based on AR manometry findings.

Our center uses two types of systems for AR manometry testing (Figure 1). The first is a portable manometry device (mCompass by Medspira, Minneapolis, MN, USA) used in clinics for bedside testing. This system consists of a tablet computer, wireless manometer, disposable catheter with four small radially oriented balloons that span the anal canal, and a single large rectal balloon. The second is a more typical computer tower system (Solar GI by Laborie) consisting of a solid-state HR AR manometry catheter, with studies typically performed in an outpatient procedure center. The catchment area for our center encompasses multiple states, meaning many patients travel long distances for clinic appointments or procedures. Having the ability to perform testing in the office reduces economic and time strains on families during the diagnostic work-up for colorectal disorders. 

Patients evaluated for disordered defecation had previously failed routine management with a minimum of a daily oral stool softener under the direction of a pediatric gastroenterologist, pediatric surgeon, or comprehensive care pediatric provider with experience managing defecation disorders. The electronic medical record was reviewed, and demographics, diagnostic findings, and clinical outcomes were described. Data are summarized as median and interquartile range (IQR) for non-parametric data or mean ± standard deviation unless otherwise stated. Categorical non-parametric data were compared using a two-tailed chi-squared test. Continuous parametric data were compared with Welch’s *t*-test using GraphPad Prism version 10.2.3 for Windows, GraphPad Software, Boston, MA, USA.

### 4.2. Treatment Protocol

**Figure 3:**
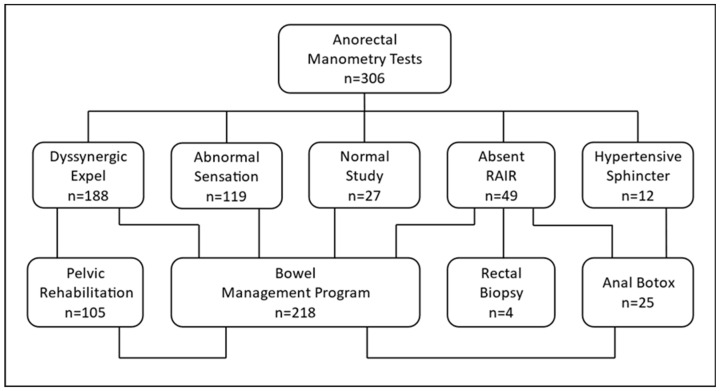
Management strategy from anorectal manometry testing results. Basic management strategies for anorectal (AR) manometry testing based on study results. The number (n) of patients in each group from our cohort is highlighted. Patients could have more than one abnormality and be treated accordingly. Rectoanal inhibitory reflex (RAIR).

AR manometry study results helped guide the management of patients with disordered defecation (Figure 3). Children with severe constipation but normal AR manometry results were typically treated with high-dose stimulant laxatives or large-volume enemas (LVEs) as part of a formal Bowel Management Program, which has been described previously [22]. The decision to treat with high-dose stimulant laxatives or LVEs was made using joint decision making with caregivers, patients, and medical providers. Factors considered when deciding treatment intervention included the failure of prior medical therapies, ability to tolerate oral medications or enema therapy, caregiver and patient preference, and results of radiographic and manometry testing. Children with a massively dilated rectum identified with water-soluble contrast enema or with markedly abnormal sensation thresholds on manometry were often treated with large-volume enema therapy without an initial trial of high-dose stimulant laxatives. Children with dyssynergic stooling were referred to physical therapy for PR. Patients unable to adequately participate in PR due to young age, developmental delay, anxiety, or neurologic impairment were not referred. Some patients lacking a RAIR without a clear explanation, such as a known neurogenic abnormality, were referred for surgical biopsy. Some children who exhibited an abnormal cough reflux or other red flag symptoms such as refractory response to interventions or lower extremity weakness had magnetic resonance imaging (MRI) of their lumbosacral spine to screen for a tethered cord. 

## 5. Results 

A total of 297 patients (male = 169, 56.9%) with a median age of 9.4 years (range 0–19, IQR 5.8 years) underwent AR manometry testing. Nine patients underwent repeat testing for a total of 306 studies. Of these, 74.8% (n = 229) of studies were performed using a portable AR manometry system (mCompass, Medspira), while the remaining 25.2% (n = 77) utilized a solid-state HR AR manometry system (Solar, Laborie, Portsmouth, NH, USA). A total of 12.1% (n = 37) of the studies were performed with sedation. Indication for testing was primarily severe constipation and/or fecal soiling, with 82.2% (n = 244) of patients having suspected functional constipation (Table 1). Fecal soiling was present in 59.3% (n = 176) of children at presentation. Other diagnoses included rectal prolapse (5.7%, n = 17), anorectal malformation (5.1%, n = 15), HD post repair (4.6%, n = 14), spinal anomalies like tethered cord (2%, n = 6) or spina bifida (1%, n = 3), and anal trauma (1.7%, n = 5). A summary of manometric findings by diagnosis is outlined in Table 2. 

Anal sphincter resting pressures were lower in patients with repaired ARM and fecal soiling compared to patients with functional constipation and fecal soiling (31 ± 13.5, 55.4 ± 11.4, mmHg, *p* < 0.0001). Resting pressure was lower in patients with functional constipation with fecal soiling compared to those with functional constipation without fecal soiling (55.4 ± 11.4, 63.3 ± 11.6, mmHg, *p* < 0.0001). Patients with rectal prolapse had lower anal sphincter resting pressure than patients with functional constipation alone (48.6 ± 13, 58 ± 12.1, mmHg, *p* = 0.007).

Maximum anal sphincter squeeze pressures were lower in patients with repaired ARM compared to patients with functional constipation (75.5 ± 25, 136.7 ± 45.5, mmHg, *p* < 0.0001). Maximum squeeze pressure was lower in patients with functional constipation with fecal soiling compared to those with functional constipation without fecal soiling (127.2 ± 42.4, 152.1 ± 51, mmHg, *p* = 0.01). Maximum squeeze pressure was also lower in patients with rectal prolapse compared to functional constipation alone (112.3 ± 38.4, 138.6 ± 46, mmHg, *p* = 0.02).

Sensation thresholds were measured in 89% (n = 239) of awake patients. A total of 13.8% (n = 37) of studies had stool urgency with rectal balloon volumes > 180 mL. There was no difference in initial sensation volumes during rectal balloon inflation in patients with functional constipation with fecal soiling compared to those without fecal soiling (61.9 ± 39.7, 51.6 ± 40.4, mL, *p* = 0.13). Similarly, there was no difference in reported stool urgency sensation thresholds between patients with functional constipation with fecal soiling compared to those with functional constipation without fecal soiling (127.6 ± 41.1, 116.9 ± 45.4, mL, *p* = 0.052).

Following testing, 65% (n = 194) of patients were treated with high-dose laxatives such as senna or bisacodyl as initial therapy. A total of 34% (n = 101) were either initially or subsequently treated with LVEs as part of our Bowel Management Program [22]. A total of 30% (n = 30) of the patients using LVEs had an appendicostomy or cecostomy for antegrade enema therapy. A total of 88.1% (n = 155/176) of patients with soiling at presentation had follow-up after initial treatment, with 21 patients having no follow-up data. A total of 78.1% (n = 121/155) of patients who were previously soiling were continent following treatment with a bowel management program. A total of 21.9% (n = 34) continued to soil to some degree, although 44.1% (n = 15) of those still soiling had significant improvement. A total of 8% (n = 25) had intrasphincteric anal abotulinum toxin A injections for retentive behaviors or high anal sphincter tone. 

A RAIR was absent in 16% (n = 49/306) of all tests. Of the patients lacking a RAIR, 49% (n = 24) had a known underlying anatomic or neurogenic cause of the absent RAIR, such as post-surgical HD (n = 14) or an anorectal malformation (n = 10). They underwent testing to better characterize rectal sensation and anal sphincter tone due to persistent soiling or constipation. A total of 16.3% (n = 8) had a RAIR present on repeat AR manometry testing under sedation. Rectal biopsy was performed in 8.3% (n = 4) patients with an absent RAIR, all of which demonstrated ganglion cells and supported a diagnosis of anal achalasia. No new diagnoses of HD were made. A total of 10% (n = 5) of patients with an absent RAIR was attributed to inadequate balloon volume inflation in the setting of a massively dilated rectum. These patients responded clinically to oral laxatives alone, and additional work-up was not pursued. A total of 8% (n = 4) had no follow-up data. The presence of a large amount of stool in the rectal vault in 2% (n = 1) of patients was thought to have contributed to the absent RAIR. A total of 4% (n = 2) of patients without a RAIR was attributed to prior anal canal trauma. 

PR with a physical therapist who had specialized training in pelvic therapy was a common treatment modality, with 35% (n = 105) of patients completing one or more sessions of PR. Reasons for PR referral included dyssynergic stooling, recurrent rectal prolapse, refractory symptoms despite optimized medical management, or hypotensive anal resting or squeeze pressures (Table 3). Reasons for not referring to PR included resolution of symptoms with medical intervention alone, developmental delay impeding cooperation with therapy, insurance coverage limitations, and exceptional distance for the family to travel for a physical therapy provider. A total of 69.5% (n = 73/105) of the patients who participated in PR were soiling prior to therapy. A total of 47% (n = 73/155) of the patients with fecal soiling who had long-term follow-up participated in PR, while 53% (82/155) had no PR. A total of 79.5% (n = 58/73) of the patients who were soiling and participated in PR were continent with continued bowel management after PR. By comparison, 76.8% (n = 63/82) of patients with soiling treated with bowel management alone without PR were continent (Table 3). There was no statistical difference between patients with fecal soiling treated with bowel management alone and those treated with bowel management and PR (*p* = 0.69). 

Dyssynergic stooling patterns were identified in 63% (n = 188) of all patients. Defecatory dynamics were performed in 81% (n = 249) of all studies. A total of 72.2% (n = 127/176) of patients with fecal soiling had dyssynergia. A total of 50% (n = 94) of patients with dyssynergia completed some PR. A total of 89.5% (n = 94/105) of patients who participated in PR had dyssynergia. A total of 88.1% (n = 112/127) of patients with fecal soiling and dyssynergia had a long-term follow-up, while 15 patients had no follow-up. A total of 57.1% (n = 64/112) of patients with fecal soiling and dyssynergia participated in PR. A total of 84.3% (n = 58/64) of patients with fecal soiling and dyssynergia were continent with PR and continued bowel management. In comparison, 77.1% (n = 37/48) of patients with fecal soiling and dyssynergia that did not have PR were continent with bowel management alone. There was no statistical difference between patients with dyssynergia and fecal soiling treated with bowel management alone and those treated with bowel management and PR (*p* = 0.33). 

There were no major complications during the study period associated with AR manometry testing. Minor complications included anxiety prior to starting the study and some discomfort with the insertion of the manometry catheter. Nearly all children reported that the study was more comfortable than they had imagined. There were minor problems with the manometry equipment. With regards to the portable manometry device, the air-filled rectal balloon on the disposable catheter burst during sensation testing in one patient, with no resulting discomfort or injury to the patient. The wireless manometer required repair by the company on two occasions. 

## 6. Discussion

This study highlights a retrospective review of 306 AR manometry tests performed over six years in a single pediatric colorectal center. Yates et al., in a systematic review of 227 publications on AR manometry testing in pediatric patients, found a male predominance in 62% of studies [46]. This was also reflected in our population, which was 57% male. 

A prospective study by Banasiuk et al. found AR manometry to be a safe and useful tool in guiding post-surgical care of pediatric patients with HD or anal atresia experiencing protracted symptoms, particularly for identifying hypotensive anal sphincter resting or squeeze pressures [47]. We similarly found AR manometry helpful in the management of persistent symptoms in patients with ARM or HD after repair. Specifically, findings of abnormal anal sphincter resting and squeeze pressures, dyssynergic stooling patterns, or abnormal rectal sensation thresholds were used to guide management decisions such as referral to PR or long-term need for continence enema therapy.

El-Shawbrawi et al., in a prospective study of 50 children aged 6–14 years with functional constipation, found rectal sensation parameters were higher in patients with fecal soiling [6]. In contrast, we found no difference between rectal sensation thresholds for initial sensation and stool urgency in patients with functional constipation and fecal soiling compared to those without fecal soiling. This may be a reflection of the complex behavioral and medical factors that contribute to fecal soiling in patients with functional constipation. 

Banasiuk et al., in a prospective study, described dyssynergia in 69.3% of 205 children with functional constipation who had AR manometry testing [48]. Dyssynergia was present in 72% of patients in our study with functional constipation and 63% of our total cohort. Adult studies have described dyssynergia in 27–59% of adults with persistent constipation [17]. 

Morera et al. described a retrospective cohort of 219 pediatric patients with functional constipation who had AR manometry and colonic manometry testing over a 7-year period [49]. Their study focused mainly on involuntary AR manometry parameters such as resting pressure and the presence of a RAIR. Defecation dynamics were tested in only 24% of patients, and defecatory sensation thresholds in 19%. We tested defecation dynamics in 81% and sensation thresholds in 78% of studies. They found no association between AR manometry findings and therapeutic response to bowel management, with similarly favorable outcomes after treatment in 72% of those with normal and 70% of those with abnormal AR manometry results. In our study, the presence of abnormal dyssynergic stooling patterns did not predict the persistence of fecal soiling after bowel management in patients with functional constipation. After typical bowel management, 76.8% of patients with fecal soiling alone and 77.1% of those with fecal soiling and dyssynergia had resolution of fecal soiling. A total of 84.3% of patients with fecal soiling and dyssynergia had resolution after treatment with PR plus bowel management, but this was not significantly different from patients treated with bowel management alone.

Finally, Bharucha et al. found the use of the portable AR manometry device in adult patients to be safe and correlate with solid-state and water-perfused HR AR manometry parameters [8]. Based on the 229 pediatric studies performed in our center using the portable AR manometry device, we have also found this testing modality to be a safe and useful option with similar utility to solid-state studies. To our knowledge, this is the first study highlighting a multi-year experience using portable AR manometry in pediatric patients.

As this is a retrospective study, there are several limitations. We did not examine the therapeutic outcomes of patients treated in our center who did not have AR manometry testing. There may be a selection bias for patients with severe or longer-lasting symptoms influencing outcomes, as these patients may be more likely to have AR manometry testing. Patients were not randomized into therapy groups with and without PR, making it difficult to determine the efficacy of pelvic rehabilitation compared to medical therapy alone. It is impossible to know which of the patients who were successfully treated in the pelvic rehabilitation group would have continued to soil without this therapy. Additionally, 12% (n = 21) of patients with fecal soiling in the medically-only treated group were lost to follow-up and may have continued to soil. 

## 7. Conclusions

AR manometry is a diagnostically useful testing modality in pediatric patients with defecation disorders. AR manometry proffers the ability to screen for HD and disordered defecation dynamics in a minimally invasive manner. This is the first study describing the use and multi-year experience using portable AR manometry testing in pediatric patients. As a secondary outcome, our group has found pelvic rehabilitation to be a helpful adjunct therapy in refractory cases of constipation, as well as persistent rectal prolapse. The efficacy may be due in part to the frequent nature of follow-up appointments, which helps reinforce compliance with therapies and behavior modification. AR manometry is a feasible testing modality to guide the management of pediatric patients with defecation disorders.

## Figures and Tables

**Table 1:** Demographics and indication for anorectal manometry testing.

Demographics	Total Patients *n* = 297
Age in years, median (range)	9.4 (0.1–19.5)
Sex, *n* (%)	
Male	169 (57)
Female	128 (43)
Ethnicity, *n* (%)	
White	265 (89.2)
Hispanic	23 (7.7)
Black	6 (2)
Native Hawaiian or Pacific Islander	2 (0.7)
Other	1 (0.3)
Diagnoses at Testing, *n* (%)	
Functional Constipation	244 (82.2)
Fecal Soiling	177 (59.6)
Rectal Prolapse	17 (5.7)
Anorectal Malformation	15 (5.1)
Hirschsprung Disease Post Repair	14 (4.7)
Tethered Cord	6 (0.02)
Anal Trauma	5 (0.02)
Spina Bifida	2 (0.01)
Other	1 (0.01)

Demographics including age, sex, and ethnicity of patients who had anorectal manometry testing. The diagnoses at the time of testing are highlighted, with “Other” being a patient with perianal Crohn’s disease who required anoplasty and diversion.

**Table 2:** Summary of sedated and awake anorectal manometry test results based on underlying diagnosis.

Manometry Findings *n* (%)	All Studies 306	Constipation 244 (82.2)	Fecal Soiling 176 (59.3)	Rectal Prolapse 17 (5.7)	ARM 15 (5.1)	HD 14 (4.6)	Spine Anomaly 9 (3)	Anal Trauma 5 (1.7)	Anal Achalasia 4 (1.3)
Sedated	37	30 (81.1)	20 (54.1)	-	-	-	-	-	-
Resting Pressure (mmHg)	33.7 ± 11.9	33.3 ± 12.2	34.1 ± 11.9	-	-	-	-	-	-
Absent RAIR	1 (3)	1 (6)	-	-	-	-	-	-	-
Awake	269	215 (79.9)	162 (60.2)	17 (6.3)	15 (5.5)	14 (5.2)	9 (3.3)	5 (1.8)	4 (1.5)
Portable Air Charged Catheter	229 (85.1)	186 (86.5)	150 (92.6)	17 (100)	12 (80)	13 (92.9)	8 (88.9)	1 (20)	-
HR Solid State Catheter	40 (14.9)	29 (13.5)	12 (7.4)	-	3 (20)	1 (7.1)	1 (11.1)	4 (80)	4 (100)
Resting Pressure (mmHg ± StDev)	55.5 ± 14.5	58 ± 12.1	53.2 ± 13.5	48.1 ± 13	31 ± 13.5	49.9 ± 11.9	51.6 ± 15	37 ± 31	72.8 ± 23.6
<30 mmHg (hypotensive)	15 (5.6)	4 (1.9)	10 (6.2)	1 (5.9)	9 (60)	-	2 (22.2)	2 (40)	-
>80 mmHg (hypertensive)	12 (4.4)	9 (4.2)	5 (3.1)	-	-	-	-	1 (20)	2 (50)
Absent RAIR	49 (18.2)	19 (8.8)	38 (23.5)	2 (11.8)	10 (66.7)	14 (100)	-	2 (40)	4 (100)
Max Anal Squeeze (mmHg ± StDev)	130.1 ± 46.1	136.7 ± 45.4	127.2 ± 42.6	112.3 ± 38.4	75.5 ± 25	127.5 ± 28.9	96.4 ± 39.1	133.8 ± 123.2	135.3 ± 26.8
Dyssynergia	188 (69.9)	155 (72.1)	127 (78.4)	13 (76.5)	11 (78.6)	9 (64.3)	6 (66.7)	1 (20)	3 (75)
Rectal Sensation									
Initial Sensation < 20 mL	8 (3)	4 (1.9)	2 (1.2)	2 (11.8)	1 (6.7)	-	-	2 (40)	1 (25)
Initial Sensation > 70 mL	56 (20.8)	47 (21.9)	43 (26.5)	-	2 (13.3)	3 (21.4)	2 (22.2)	-	-
Desire to Defecate < 40 mL	26 (9.7)	13 (6)	15 (9.3)	-	5 (33.3)	1 (7.1)	-	3 (60)	2 (50)
Desire to Defecate > 150 mL	20 (7.4)	16 (7.4)	13 (8)	-	2 (13.3)	-	2 (22.2)	-	-
Stool Urgency < 60 mL	14 (5.2)	5 (2.3)	7 (4.3)	-	3 (20)	1 (7.1)	-	3 (60)	-
Stool Urgency > 180 mL	37 (13.8)	31 (14.4)	24 (14.8)	1 (5.9)	1 (6.7)	1 (7.1)	1 (11.1)	-	-

All sedated studies were performed using a high-resolution (HR) solid-state catheter. Of note, 9 of the 297 patients had a repeat study, for a total of 306 studies. Results are presented as the number of patients with a specific finding (percentage of each diagnosis with that finding) or as mean ± standard deviation (StDev). Anorectal malformation (ARM), post-repair Hirschsprung disease (HD), rectoanal inhibitory reflex (RAIR).

**Table 3:** Pelvic rehabilitation, dyssynergia, and fecal soiling.

Pelvic Rehabilitation, Dyssynergia & Fecal Soiling		Total PR Patients *n* = 105	
Pre-PR Diagnoses, *n* (%)			
Dyssynergia	94 (89.5)		
+Fecal Soiling	64 (59.8)		
+Rectal Prolapse	10 (9.5)		
+Rectal Hyposensitivity	12 (11.4)		
+Rectal Hypersensitivity	2 (1.9)		
+Hypotensive AS	2 (1.9)		
Fecal Soiling	9 (8.6)		
Hypotensive AS	1 (1)		
Constipation	1 (1)		
Fecal Soiling ± PR, *n* (%)	Total	+PR	−PR
Soiling Pre-Treatment	155	73 (47.1)	82 (52.9)
+Dyssynergia	112 (81.9)	64 (41.3)	48 (31)
Continent Post-Treatment	121 (78.1)	58 (79.5)	63 (76.8)
+Dyssynergia	91 (81.2)	54 (84.3)	37 (77.1)
Fecal Soiling Treatment ± PR, *n* (%)			
High Dose Laxative	49 (31.6)	27 (37)	22 (26.8)
LVE	79 (51)	33 (45.2)	46 (56.1)
ACE	18 (11.6)	10 (13.7)	8 (9.8)
Other	9 (5.8)	3 (4.1)	6 (7.3)

Overview of pre-treatment diagnoses of patients that participated in pelvic rehabilitation (PR), treatment modalities, and continence outcomes of patients with fecal soiling and dyssynergia. Results are presented as the number and percentage of patients (*n*, %) within a diagnosis or treatment group. The “Other” treatment groups of patients with fecal soiling included stool softeners or no medications. There was no statistical difference in continence rates between the PR and non-PR treatment groups of patients with fecal soiling (*p* = 0.69) or with fecal soiling and dyssynergia (*p* = 0.33). A total of 155 of 176 patients with fecal soiling at presentation had follow-up data. A total of 112 of 127 patients with fecal soiling and dyssynergia had follow-up data. Patients without follow-up data were excluded. Anal sphincter (AS), retrograde large-volume enema (LVE), antegrade continence enema (ACE).

## Data Availability

The data presented in this study are available upon request from the corresponding author due to privacy limitations.
